# EGFR signaling pathway and related-miRNAs in age-related diseases: the example of miR-221 and miR-222

**DOI:** 10.3389/fgene.2012.00286

**Published:** 2012-12-07

**Authors:** Ana L. Teixeira, Mónica Gomes, Rui Medeiros

**Affiliations:** ^1^Molecular Oncology Group, Portuguese Institute of Oncology of PortoPorto, Portugal; ^2^Abel Salazar Institute for the Biomedical Sciences, University of PortoPorto, Portugal; ^3^LPCC, Research Department-Portuguese League Against Cancer (NRNorte)Porto, Portugal; ^4^CEBIMED, Faculty of Health Sciences of Fernando Pessoa UniversityPorto, Portugal

**Keywords:** EGFR, miRNAs, miR-221/222, age-related diseases, cancer, neurodegenerative diseases

## Abstract

Presently, neurodegenerative diseases and cancer are the most clinically problematic age-related diseases worldwide. Although being distinct disorders, their developments share common cellular mechanisms. Oncogenesis and neurodegeneration arise from the deregulation of signaling pathways, as a consequence of the resulting imbalance in cellular homeostasis. The epidermal growth factor receptor (EGFR) belongs to an important cellular signaling pathway, which regulates proliferation, differentiation, cell cycle and migration. As transcriptional targets of EGFR, the microRNAs-221/222 (miR-221/222) are important expression regulators. Dysfunctions in their networks are associated with cellular disruptions. The transcriptional activation of these microRNAs (miRNAs) seems to be involved in cell cycle, apoptosis, metastization, and in the acquisition of resistance to therapies. The up-regulation of miR-221/222 is associated with increased expression levels of matrix metalloproteinases (MMPs) and repression of cell cycle inhibitors, which are key molecules in oncogenesis and neurodegeneration processes. The interaction loop between proliferative signaling pathways and miRNA expression could reveal new targets for controlling the molecular behavior of age-related diseases.

## Introduction

Presently, neurodegenerative diseases (NDs) and cancer are the two most clinically problematic age-related diseases with a relevant impact on public health worldwide, with incidence rates growing over the years alongside with the aging of the populations (Du and Pertsemlidis, 2011).

Cancer and ND are a complex group of different diseases, with different etiologies, treatments and prognosis. Although ND and cancer are two distinct clinical disorders, where the high cellular division rate of cancer cells contrast with the low cellular divisions observed in neurons, it has been hypothesized that oncogenesis and neurodegeneration may share common cellular mechanisms. One of those mechanisms is the deregulation of proliferative signaling pathways (Morris et al., 2010).

Several environmental factors, like viral infections and exposure to certain toxic compounds, might be involved in oncogenesis and neurodegeneration initiation. These factors could be responsible for mutations in multiple genes involved in cell cycle progression, DNA repair, and cell proliferation leading to cellular deregulation.

Cells usually have a tight control over their signaling pathways, resulting in precise gene expression. Deregulation of these pathways can have dramatic effects on the expression of target downstream genes and protein formation, resulting in cellular homeostasis imbalance. Cellular homeostasis is normally regulated by the concerted actions of both mitogenic growth signals and anti-proliferative signals. In fact, some authors suggest that aberrant growth and differentiation are caused by an inappropriate cellular microenvironment (Long et al., 2005). Functional genetic polymorphisms, with the ability to modulate cellular microenvironment, could influence the carcinogenic processes. For instance, changes in circulating levels of epidermal growth factor (EGF) and transforming growth factor beta 1 (TGFβ1), due to functional genetic variants, could influence the risk to develop prostate and lung cancer, respectively (Teixeira et al., 2008, 2011). Several studies showed that these genetic alterations, with an impact on the cellular microenvironment, could also be involved in the acquisition of resistant phenotypes to different antineoplastic therapies. Moreover, changes in the proliferative epidermal growth factor receptor (EGFR) pathway influence the response to androgen deprivation therapy (in advanced cases of the prostate cancer) and can modulate cancer progression in different cancer models (Wang et al., 2007; Teixeira et al., 2009; Costa et al., 2011).

Deregulation of signaling pathways is a hallmark which often occurs in malignant diseases. The EGFR transactivation stimulates a network of cytoplasmatic transduction molecules, leading to a transcriptional activation and consequent modulation of a wide variety of cellular functions, including cell proliferation, migration, adhesion, and differentiation. The up-regulation of this pathway has been found in several tumors, including non–small cell lung cancer (NSCLC), head and neck carcinoma, gliomas, and colorectal carcinoma (Chung et al., 2006; Coldren et al., 2006). This suggests that EGFR overexpression is an important event in cancer development and progression. The activation of the EGFR pathway is also responsible for the transcriptional activation of specific microRNAs (miRNAs), which are a new class of biomarkers involved in gene regulation (Shah and Calin, 2011; Stinson et al., 2011).

MiRNAs are a family of endogenous and short non-coding RNAs (20–25 nucleotides) that exert their effect by negatively regulating gene expression through one of the two mechanisms: mRNA degradation or translational suppression. MiRNAs play an important role in different biological processes, such as cell proliferation, cell growth, apoptosis, signal transduction, cell cycle, and neurogenesis (Shah et al., 2009; Shah and Calin, 2011). Changes in miRNA regulation networks can cause disruptions of the normal cellular activity leading to disease development. The tight control necessary for cellular homeostasis, evolutionarily provided the cell with mechanisms for precise gene regulation. Recently, miRNAs emerged as an important class of gene expression regulators involved in cancer and neurodegenerative pathologies (Cooper et al., 2009).

The widespread deregulation of miRNAs in all types of tumors, may allow us to consider that their expression signature is a useful diagnostic and prognostic tool. MiRNA expression analysis has confirmed that specific miRNAs show differential expression patterns between normal and tumor tissues. For example, miR-10b is specifically overexpressed in metastatic breast cancer, but downregulated in primary breast tumors (Iorio et al., 2005). In the process of neuron deterioration, studies performed by Kim and co-workers showed that alterations in miRNA networks of the brain may result in neurodegenerative disorders (Kim et al., 2007). In addition, Ghelani and co-workers observed that the deregulation of the expression of specific miRNAs could be associated with several types of neurodegenerative disorders, the downregulation of miR-9 and miR-107 were associated with Alzheimer's disease (AD), due to their impact on the modulation of insulin resistance and innate immunity pathways (Shioya et al., 2010; Ghelani et al., 2012; Saito and Saito, 2012). MiRNAs also demonstrate a unique pattern of regional and subcellular localization in the brain: for example, miR-221/222 are preferentially found in the hippocampus, while miR-195, miR-497, and miR-30b are found in the cerebellum (Feng and Feng, 2011).

Several studies have shown an interesting interaction loop between growth factor activation of the EGFR pathway and the transcriptional activation of specific miRNAs (Hayashi et al., 2009; Avraham et al., 2010). Studies performed by Avraham and co-workers showed that after EGF stimulation, cells initiated a coordinated transcriptional program of miRNAs and transcription factors involved in a rapid induction of oncogenic transcription factors, such as c-FOS, encoded by immediate early genes (Avraham et al., 2010). Hayashi and co-workers observed in a fetal murine submandibular salivary grand (SMG) model (useful in the study of organogenesis, differentiation, proliferation, and epithelial-mesenchymal interaction) that different miRNA profiles were expressed specifically at different EGF concentrations *in vitro* (Hayashi et al., 2009; Avraham et al., 2010).

**Figure 1:**
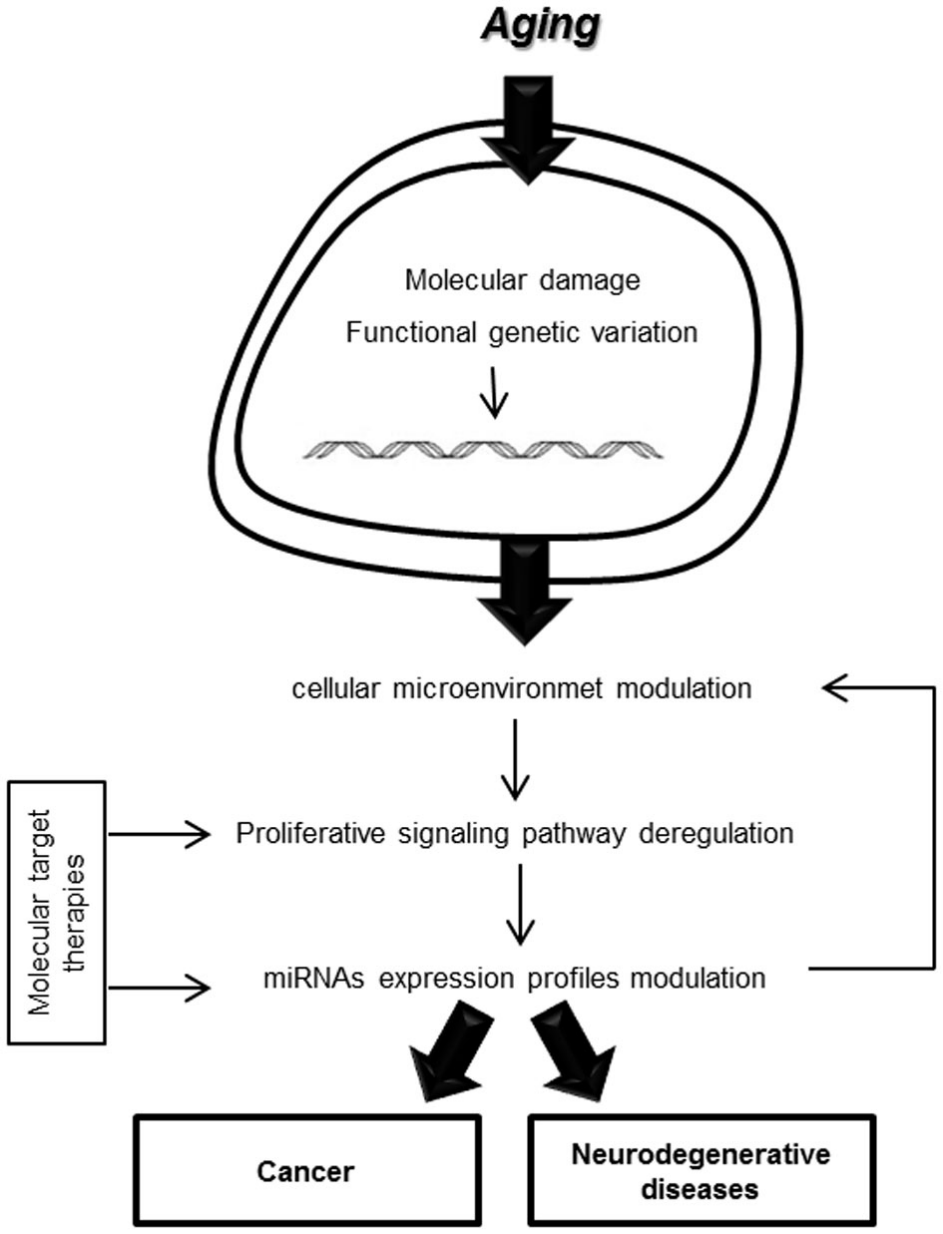
**Cellular microenvironment modulation in aging processes**.

The interaction link between proliferative signaling pathways and miRNA expression profiles involved in oncogenesis and neurodegeneration could reveal interesting molecular targets for the development of new therapies that would improve the management of age-related diseases (Figure 1).

## The role of the EGFR signaling pathway in cancer and neurodegenerative diseases

EGFR, also known as HER-1 or ErbB-1, is a transmembrane glycoprotein with tyrosine kinase activity. This receptor belongs to the ErbB family of receptors kinases and regulates relevant cellular processes, including proliferation, differentiation, cell cycle progression, and migration. In addition, EGFR is involved in the pathogenesis and maintenance of several human cancers of epithelial origin (Webster et al., 2009).

In a normal tissue, the ligand EGF binds to the EGFR inducing the dimerization of one or several members of the EGF receptor family (ErbB 1–4). This process activates several tyrosine kinases, and other downstream signaling molecules, promoting gene transcription in the cell's nucleus (Wang et al., 2007).

In cancer cells, EGFR ligand levels are frequently elevated and EGFR itself is commonly overexpressed (Vicentini et al., 2003). This overexpression, as well as structural genetic alterations, has been reported in many malignancies including breast cancer, prostate cancer, NSCLC, gliomas, and also been associated with poor prognosis (De Muga et al., 2010). EGFR mutations are more frequently reported in lung cancer, compared with prostate and colorectal cancer, and very uncommon in head and neck, pancreas, esophagus and breast cancers (Lee et al., 2005, 2007; Kwak et al., 2006; Sharma et al., 2007; Sibilia et al., 2007). However, other variations, such as single nucleotide polymorphism in the *EGFR* gene, can also modulate the microenvironment. For example, the G>T transition in the promoter position −260 of *EGFR* increases the promoter's activity (Liu et al., 2005) (Table 1).

**Table 1:** **Alterations of components of the EGFR signaling pathway in age-related diseases**.

**Age-related disease**	**EGF/EGFR signaling pathway**
Prostate cancer	EGFR overexpressed and moderately mutated; ligands frequently overexpressed; EGF/EGFR genetic polymorphisms
Breast cancer	EGFR overexpressed and rarely mutated; EGF/EGFR genetic polymorphisms
Lung cancer	EGFR frequently mutated; EGF/EGFR genetic polymorphisms
Glioma	EGFR frequently overexpressed; ligands overexpressed; EGF/EGFR genetic polymorphisms
Colorectal cancer	EGFR frequently overexpressed and moderately mutated; ligands overexpressed; EGF/EGFR genetic polymorphisms
Head and neck cancer	EGFR frequently overexpressed and rarely mutated; EGF/EGFR genetic polymorphisms
Pancreatic cancer	EGFR overexpressed and rarely mutated; EGF/EGFR genetic polymorphisms
Esophagus cancer	EGFR overexpressed and rarely mutated; EGF/EGFR genetic polymorphisms
Alzheimer's disease	EGFR and ligands overexpressed
Parkinson's disease	EGFR overexpressed in chronic Parkinsonian syndromes

Nowadays, EGFR *status* is a prognostic tool in several cancers, indicating poor survival, more aggressive behavior, increased risk of invasion/metastasis, and resistance to antineoplastic therapies (Press et al., 2008). In fact, this pathway has been proposed to be involved in hormone-resistant prostate cancer development, as an alternative proliferative pathway in the absence of androgens during androgen deprivation therapy, conferring a poor prognosis to the patients, with limited therapeutic options (Attar et al., 2009).

The important role of the EGFR signaling pathway in oncogenesis made it a good candidate for targeted cancer therapy. Early studies demonstrated that targeting the catalytic domain of EGFR, using tyrosine kinase inhibitors (TKIs), had an anticancer effect (Fry et al., 1994; Bos et al., 1997). These TKIs inhibit the growth of cancer cells by inducing cell-cycle arrest and/or apoptosis. Several anti-EGFR strategies that target different components of the EGFR-pathway have been developed in different cancer models (Amit et al., 2007). However, anti-EGFR therapies are associated with some side-effects, such as skin toxicity, due to the essential role of EGFR in normal keratinocyte biology. Nonetheless, some genetic characteristics are associated with a higher therapeutic benefit (Heist and Christiani, 2009; Dahan et al., 2011).

Alterations in *EGFR* expression levels can be also observed during neurodegeneration. Studies performed by Repetto and co-workers demonstrated that presenilin 1 (*PS1*) is a critical regulator of the EGFR pathway (Repetto et al., 2007). Mutations in *PS1* and *PS2* genes are responsible for the vast majority of early onset familial AD (Sherrington et al., 1995). Presenilin forms an active γ-secretase complex together with Nicastrin (NCT), APH-1, and PEN-2, which among other substrates cleaves the beta-amyloid precursor protein (β-APP) generating the Aβ and the β-APP intracellular domain. The *PS1* and *PS2* mutations alter the activity of the γ-secretase complex, leading to changes in the ratio of Aβ, favoring Aβ42 generation and accelerated amyloid deposition in brain, the hallmark of AD (De Strooper, 2007). However, according to Repetto and co-workers, presenilins may be involved in the modulation of signaling cell surface receptors that could alter the neuronal viability. They observed that EGFR levels were robustly increased in fibroblasts deficient in both *PS1* and *PS2* and the stable transfection of wild-type PS1 but not PS2 corrected EGFR to levels comparable to *PS*^+/+^ cells (Repetto et al., 2007). Li and co-workers demonstrated that the levels of EGFR are inversely correlated with the level of γ-secretase in fibroblasts, suggesting that the up-regulation of EGFR stimulates hyperproliferation in epithelia of mice with genetic reduction of γ-secretase (Li et al., 2007). The EGFR pathway seems to have an important role in the development of the nervous system, promoting the growth and differentiation of neural stem cells (Currais et al., 2009). However, according to Currais and co-workers, the higher levels of the EGFR specific ligand, EGF, induce neuronal death, and strong EGFR immunoreactivity has been detected in neurites surrounding neuritic plaques in AD (Currais et al., 2009). The loss of *PS1* can stimulate the activation of EGFR and β-catenin pathways, which can contribute to neurodegeneration and aberrant cell cycle re-entry (Repetto et al., 2007) (Table 1).

## The EGFR pathways and the miR-221/222 expression regulation

As a consequence of EGFR signaling activation, the transcription of several genes and regulators occurs. The EGFR pathway seems to be involved in the regulation of a panel of miRNAs essential for metastatic phenotypes and neurodegeneration.

MiRNAs arise from intergenic or intragenic genomic regions that are transcribed as long primary transcripts. The primary transcripts then undergo processing steps, which involve Drosha and Dicer enzymes, to form a mature miRNA (Calin and Croce, 2006). The mature miRNA binds to specific regions of target mRNA transcripts and destabilizes the target transcript, blocks its translation, or both. Changes in miRNA expression can contribute to oncogenesis and neurodegeneration, enhancing proliferation and leading to genomic instability, causing increased DNA damage. MiRNAs can be defined as one of the “guardians” of the genome, maintaining the genomic stability throughout the life of a cell. Major miRNA expression levels are detected in the brain; however, their expression patterns have not yet been fully described. Recent studies identified two miRNAs, miR-221 and miR-222, as downstream targets of the EGFR-RAS-RAF-MEK pathway, by the use of EGFR and MEK inhibitors, which knocked down the expression of both miRNAs and Fos-related antigen 1 (FOSL1) (Shah and Calin, 2011; Stinson et al., 2011).

Studies performed by Kumar and co-workers showed that the depletion of Dicer from various cancer cell lines increased colony formation efficiency and augmented tumor burden and aggressiveness *in vitro* (Kumar et al., 2007). In normal adult mouse skin and embryonic fibroblasts, the Dicer knock-out led to premature senescence (Shalgi et al., 2009). Dicer depletion can elicit senescence or changes in proliferation of normal or cancer cells, and can also alter the phosphorylation patterns of tau proteins before neuronal cell loss, indicating that some mechanisms of neurodegeneration might be controlled by miRNAs (Hebert et al., 2010). Studies showed the loss of midbrain dopaminergic neurons in Dicer knock-out mice in the post-mitotic midbrain, which is a Parkinson's disease (PD)-like phenotype. These observations suggest that miRNAs are essential for the terminal differentiation and/or maintenance of multiple neuron types (Srivastava et al., 2012). Psychiatric and neurological disorders, such as schizophrenia, depression, and mental health disorders also appear to be associated with changes in miRNA expression. For example, schizophrenia is associated with a global increase in miRNA biogenesis and expression in the cerebral cortex, such as the global increase in expression of miR-26b and miR-30b (Manolis and Manolis, 2012).

The up-regulation of miR-221/222 has been described in several human cancers, including glioblastoma, melanoma, hepatocellular carcinoma, kidney and bladder cancers, gastric cancer, pancreatic cancer, ovarian cancer, and prostate cancer (Negrini et al., 2009; Coppola et al., 2010). This up-regulation was also observed in circulation, as free miRNAs, in renal cell carcinoma patients compared with healthy individuals (Teixeira et al., 2012). The circulating levels of specific miRNAs are a promising strategy in the identification of cancer expression profiles predictive of treatment response, which will allow us to individualize treatments.

MiR-221/222 are also involved in the metastatic process. Their expression levels are correlated with the repression of transcriptional factors, such as the zinc finger transcription factor Trps1 (TRPS1). The repression of this factor causes an increase in the levels of the zinc finger E-box-binding homeobox 2 protein (ZEB2), which promotes a crucial step in the epithelial-to-mesenchymal transition (EMT), essential for the development of metastasis (Shah and Calin, 2011).

On the other hand, miR-221/222 seem to be involved in acquisition of resistance to antineoplastic therapies. The knockdown of miR-221/222 in the LNCaP-Abl cell line restored the response to the dihydrotestosterone (DHT) and also increased the cell line's growth response to androgen treatment, suggesting that miR-221/222 participate in androgen resistant phenotype behavior (Sun et al., 2009). Accordingly, Rao and co-workers revealed that they are also involved in acquisition of the resistance to fulvestrant, a selective estrogen receptor down-regulator (SERD): the up-regulation of these miRNAs was essential for cell growth and cell cycle progression of breast cancer resistant cells (Rao et al., 2011).

MiR-221/222 seem to have the ability to modulate cell cycle progression. Miller and co-workers demonstrated that miR-221/222 can modulate cell cycle progression, by repressing cell cycle inhibitor proteins p27/Kip1 and p57, facilitating cell proliferation and self-renewal (Miller et al., 2008). The high expression levels of miR-221/222 in glioma tissue samples have been associated with aggressiveness and poor overall survival. Their knockdown decreased the invasion capability and tumor growth and up-regulated the expression of suppressor gene metallopeptidase inhibitor 3 (TIMP3), an inhibitor of matrix metalloproteinase (MMPs) (Zhang et al., 2012). In glioblastoma cells, the overexpression of miR-221/222 promoted premature cell cycle entry, leading to cell death (Medina et al., 2008). This ability to modulate the cell cycle could help to explain the role of miR-221/222 in the pathogenesis of neurodegenerative disorders and in the apoptotic death of injured neurons. Studies performed by de las Cuevas et al. demonstrated that lymphoblasts from AD patients exhibit an enhanced stimulation of proliferation and survival compared with control individuals (De las Cuevas et al., 2003; Bartolome et al., 2007). This change in proliferative activity was associated with a high degree of phosphorylation of Akt and the downregulation of inhibitors of the G1-S checkpoint p21 and p27. The ability to control cell cycle has been considered a critical factor in preventing neurons from entering a vulnerable high risk state for neurodegeneration mechanisms.

Recently, it has been shown that the prodeath BCL-2-binding component 3 (BBC3, also known as PUMA) is one of the targets of miR-221/222. BBC3 is critical for the death of newly generated neurons in the adult brain and is also a powerful mediator of neuronal apoptosis after various insults (Harder and Libby, 2011). *In vitro* studies performed by Hamada and co-workers observed that miR-221 decreased the expression of Forkhead box O3a (Foxo3a) and apoptotic peptidase activating factor 1 (Apaf-1), both of which are known to be involved in apoptosis in PC12 cells (derived from a pheochromocytoma- rat adrenal medulla). These results suggest a possible role of this miRNA in neuronal differentiation, as protection against apoptosis (Hamada et al., 2012).

**Figure 2:**
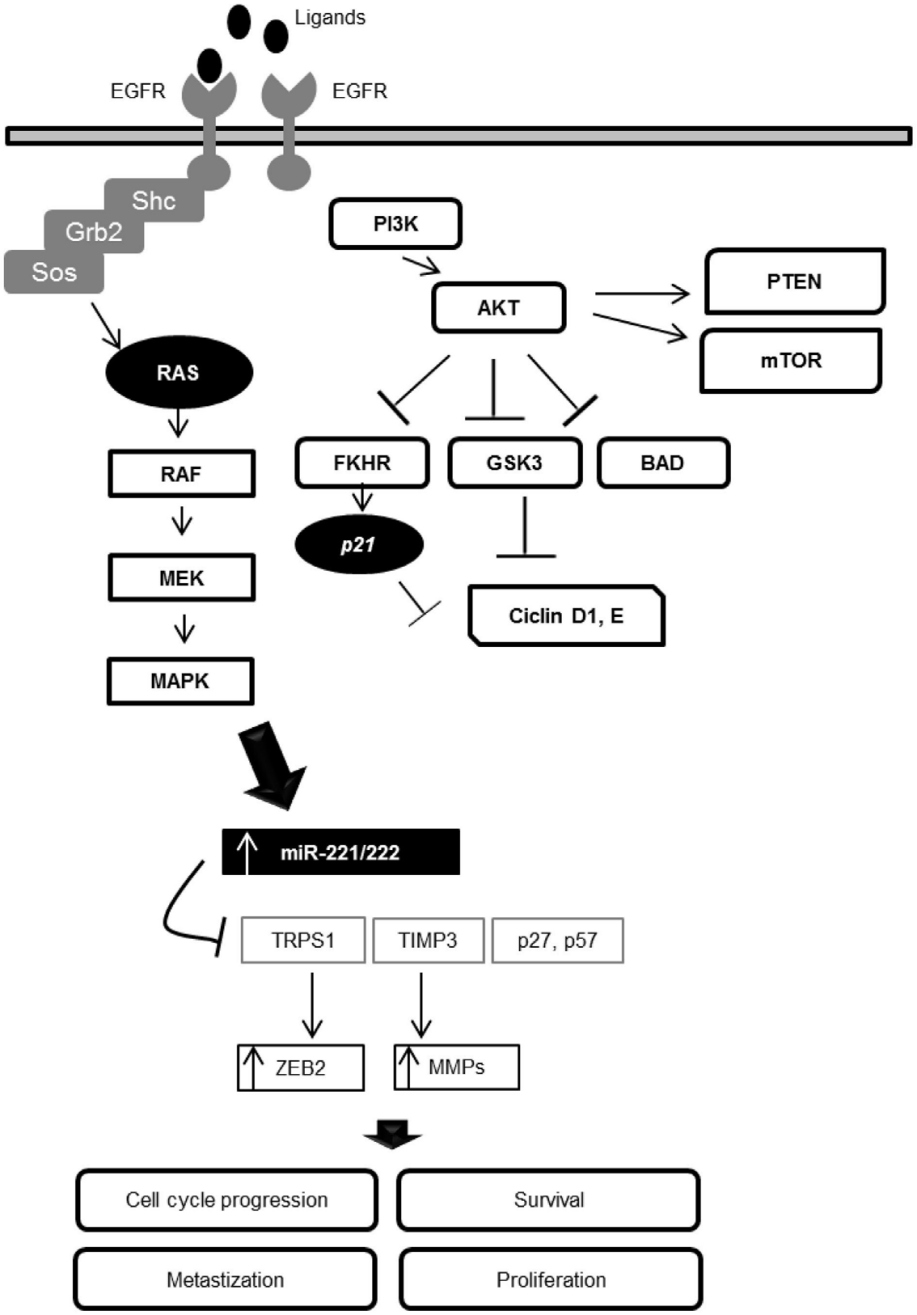
**Interaction loop between the EGFR signaling pathway and miR-221/222 expression in the modulation of cellular processes**.

In consequence of the inverse correlation of miR-221/222 and TIMP3 levels, the up-regulation of miR-221/222 is associated with the expression MMPs. It is accepted that MMPs, especially MMP3, may contribute to neurodegeneration *in vivo* by participating in neuronal apoptosis and inflammation processes (Kim and Hwang, 2011). Degradation of the inhibitory protein TIMP3 is associated with an increased expression level of MMP3 in experimental models. This elevation has been reported in several neurodegenerative disorders, such as PD, AD, white matter damage in vascular dementia, and ischemic neuronal death (Rosenberg et al., 2001a,b; Kim and Hwang, 2011) (Figure 2). The expression of MMP3 can be responsible for the coordination of an effective, rapid death, and clearance of neurons in normal conditions. However, uncontrolled production of MMP3 may result in triggering a vicious cycle, attacking neurons that are nearby and undamaged (Kim and Hwang, 2011).

MiR-221/222 are two important downstream modulators of the EGFR-RAS-RAF-MEK pathway. This interaction loop can deregulate cellular homeostasis, revealing that these two miRNAs are key EGFR pathway-effectors. Due the central role of these miRNAs in several processes and disorders, they are promising targets for the development of new molecular therapies, especially in cases where the anti-EGFR therapies demonstrate poor benefits and side effects.

### Conflict of interest statement

The authors declare that the research was conducted in the absence of any commercial or financial relationships that could be construed as a potential conflict of interest.
